# ASC- and caspase-1-deficient C57BL/6 mice do not develop demyelinating disease after infection with Theiler’s murine encephalomyelitis virus

**DOI:** 10.1038/s41598-023-38152-3

**Published:** 2023-07-06

**Authors:** Dandan Li, Melanie Bühler, Sandra Runft, Gisa Gerold, Katarzyna Marek, Wolfgang Baumgärtner, Till Strowig, Ingo Gerhauser

**Affiliations:** 1 Department of Pathology, University of Veterinary Medicine Hannover, Foundation, Bünteweg 17, 30559 Hannover, Germany; 2 Department of Biochemistry, University of Veterinary Medicine Hannover, Foundation, Bünteweg 17, 30559 Hannover, Germany; 3 Research Center for Emerging Infections and Zoonoses (RIZ), University of Veterinary Medicine Hannover, Foundation, Bünteweg 17, 30559 Hannover, Germany; 4 Wallenberg Centre for Molecular Medicine (WCMM), Umeå University, 90185 Umeå, Sweden; 5 Department of Clinical Microbiology, Virology, Umeå University, 90185 Umeå, Sweden; 6 Department for Microbial Immune Regulation, Helmholtz Centre for Infection Research, Inhoffenstraße 7, 38124 Braunschweig, Germany; 7 Hannover Medical School, Carl-Neuberg-Straße 1, 30625 Hannover, Germany

**Keywords:** Inflammasome, Infection

## Abstract

Theiler's murine encephalomyelitis virus (TMEV) induces an acute polioencephalomyelitis and a chronic demyelinating leukomyelitis in SJL mice. C57BL/6 (B6) mice generally do not develop TMEV-induced demyelinating disease (TMEV-IDD) due to virus elimination. However, TMEV can persist in specific immunodeficient B6 mice such as IFNβ^−/−^ mice and induce a demyelinating process. The proinflammatory cytokines IL-1β and IL-18 are activated by the inflammasome pathway, which consists of a pattern recognition receptor molecule sensing microbial pathogens, the adaptor molecule Apoptosis-associated speck-like protein containing a CARD (ASC), and the executioner caspase-1. To analyze the contribution of the inflammasome pathway to the resistance of B6 mice to TMEV-IDD, ASC- and caspase-1-deficient mice and wild type littermates were infected with TMEV and investigated using histology, immunohistochemistry, RT-qPCR, and Western Blot. Despite the antiviral activity of the inflammasome pathway, ASC- and caspase-1-deficient mice eliminated the virus and did not develop TMEV-IDD. Moreover, a similar IFNβ and cytokine gene expression was found in the brain of immunodeficient mice and their wild type littermates. Most importantly, Western Blot showed cleavage of IL-1β and IL-18 in all investigated mice. Consequently, inflammasome-dependent activation of IL-1β and IL-18 does not play a major role in the resistance of B6 mice to TMEV-IDD.

## Introduction

Theiler's murine encephalomyelitis virus (TMEV) is a single-stranded RNA virus of the *Picornaviridae* family^1^. The intracerebral infection of SJL mice with TO subgroup strains of TMEV (BeAn, DA) induces a biphasic disease characterized by acute polioencephalomyelitis and chronic demyelinating leukomyelitis, which represents an important animal model of human multiple sclerosis (MS)^2–4^. TMEV-induced demyelinating disease (TMEV-IDD) is initiated by oligodendrocyte apoptosis and axonal damage and aggravated by a delayed-type hypersensitivity response directed against viral antigens and later myelin components^5–7^. The virus mainly infects hippocampal and cortical neurons in the early phase of the disease but only persists in white matter glial cells and macrophages in advanced stages^8^. In contrast to SJL mice, C57BL/6 (B6) mice eliminate the virus from the central nervous system (CNS) within few weeks and do not develop TMEV-IDD^9,10^. Nevertheless, TMEV can persist in specific immunodeficient B6 mice such as interferon (IFN)-β knockout mice and even induce demyelination in their spinal cord^11^.

The proinflammatory cytokines IL-1β and IL-18 are produced as inactive precursors (Pro-IL-1β and Pro-IL-18), which are processed by caspase-1 and caspase-4/5/11 after activation of the inflammasome pathway^12–17^. Inflammasomes are vital players in innate immunity and involved in the complex host–pathogen-interactions. Typical inflammasomes represent multiprotein complexes composed of a pathogen sensor molecule such as *nucleotide binding domain and leucine-rich repeat-containing receptor* (NLR) or *AIM2-like receptor* (ALR), the adaptor molecule *apoptosis-associated speck-like protein containing a caspase activation and recruitment domain* (ASC) and the executioner caspase-1^18–21^. These molecules are mainly expressed by microglia in the brain but astrocytes and neurons as well as myeloid cells infiltrating from the periphery can produce and activate inflammasome pathway components^22,23^. Inflammasome activation prevents viral replication by inducing an inflammatory cell death program termed pyroptosis mediated by the pore-forming protein gasdermin D (GSDMD)^18,24,25^. Moreover, the inflammasome pathway has an indirect antiviral effect due to the inhibition of the immunosuppressive effects of type I IFN (IFN-I) on T cells^26,27^. Correspondingly, the NLRP3 inflammasome pathway is critical for the control of West Nile virus (WNV) infections, because it inhibits WNV replication in neurons^28,29^. Nonetheless, wild type and caspase-1-deficient mice are equally susceptible to encephalomyocarditis virus (EMCV) and vesicular stomatitis virus (VSV) infection despite the ability of the NLRP3 inflammasome to detect these viruses^30^. Interestingly, the promotion of T cell pathogenicity and CNS cell infiltration caused by inflammasome signaling contributes to the development of MS lesions^31^. Moreover, the inhibition of NLRP3 inflammasome activation by IFN-I plays a major role in the response of MS patients to IFNβ treatment^32,33^. Likewise, the NLRP3 inflammasome can induce demyelinating lesions in experimental autoimmune encephalomyelitis (EAE), an animal model of MS, because it aggravates the infiltration of inflammatory immune cells into the CNS^32^. Nevertheless, EAE can even be induced in Nlrp3^−/−^ and Asc^−/−^ mice by an aggressive immunization using a myelin oligodendrocyte glycoprotein peptide in complete Freund’s adjuvant with 300 mg of heat-killed Mycobacteria. Similar to MS, the administration of IFNβ usually ameliorates EAE but in such NLRP3 inflammasome-independent EAE this treatment is not effective^34^. *Asc*^−/−^ mice develop milder EAE lesions compared to *Casp1*^−/−^ mice due to inflammasome-independent functions of ASC, which support the survival of CD4^+^ T cells including myelin oligodendrocyte glycoprotein–specific T cells^35^. ASC also regulates the activity of the *mitogen-activated protein kinase* (MAPK) *extracellular-signal regulated kinase* (ERK) in murine and human monocytes/macrophages, antigen-specific IgG responses and chemokine and IFN-I expression independent of inflammasome activation^28,36,37^. Consequently, the different inflammasome pathway components can independently control inflammatory processes.

ASC increases serum levels of IFNα, IFNγ, IL-1β, IL-6, CCL2, CCL5, CXCL1, and immunoglobulin M (IgM) during WNV infection thereby limiting viral replication. Nevertheless, elevated and not reduced levels of IFNγ, CCL2 and CCL5 were found in the brains from WNV-infected *Asc*^−/−^ mice correlating with enhanced astrocyte activation, leukocyte infiltration and neuronal cell death^28^. Strong activation of the NLRP3 inflammasome and down-stream PGE_2_ signaling in dendritic cells and CD11b^+^ leukocytes, spleen cells and bone marrow cells of SJL mice also impairs early protective IFNγ-producing CD4^+^ and CD8^+^ T cell responses allowing viral persistence necessary for TMEV-IDD^38^. The exact role of specific inflammasome pathway components in the pathogenesis of TMEV-IDD has not been investigated so far. Therefore, the present study aimed to analyze the consequences of ASC or caspase-1 deficiency on virus elimination, CNS inflammation, and clinical outcome in TMEV-infected B6 mice. Moreover, the cytokine and IFN-I expression was analyzed in detail at 4 days post infection (dpi) due to the high number of T cells and B cells as well as peak of viral load in the brain of TMEV-infected B6 mice at this time point^39–41^.

## Results

### Clinical and histological investigation

**Figure 1:**
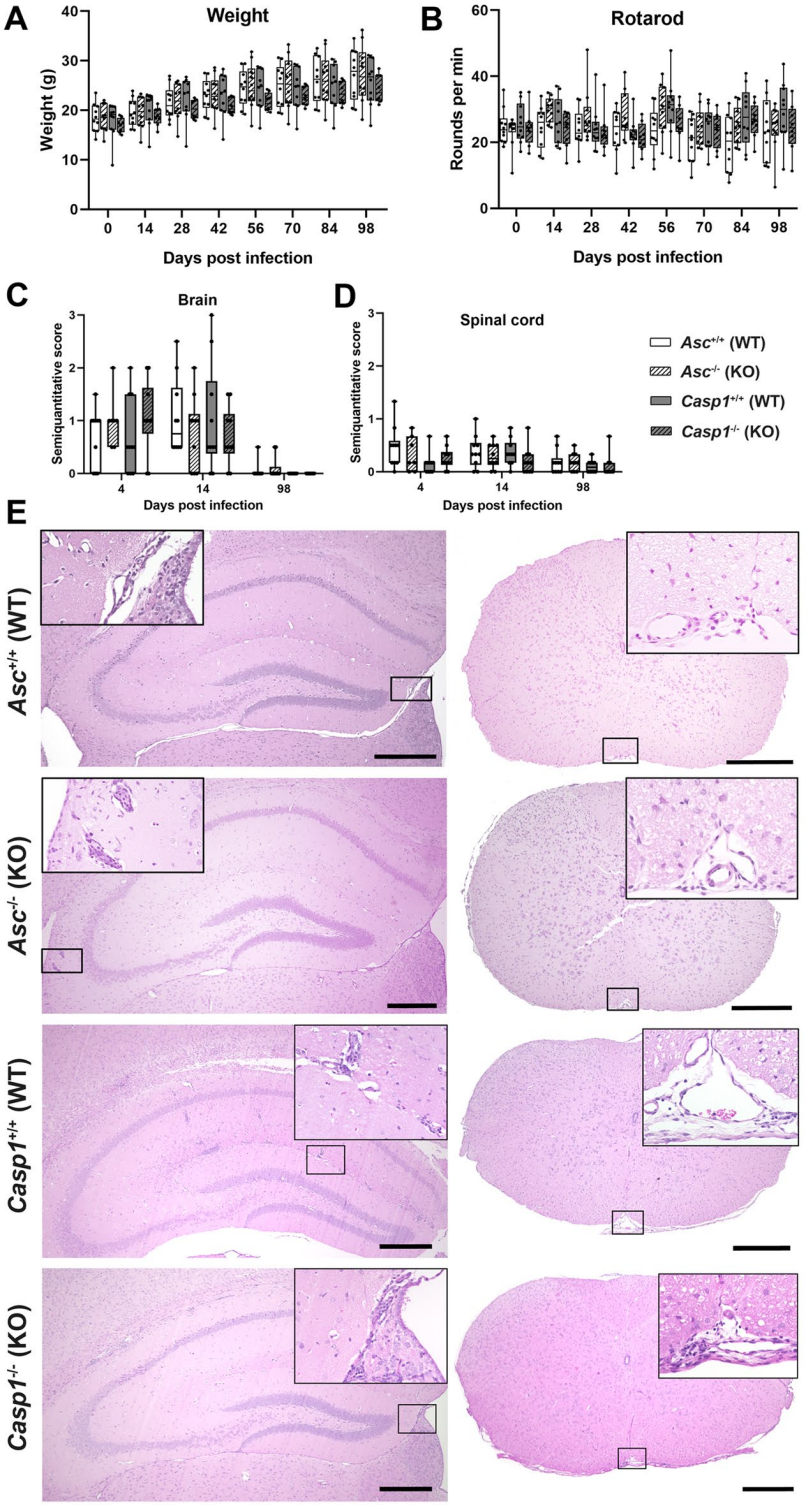
Clinical and histological data of *Asc*^−/−^ and *Casp1*^−/−^ mice (KO) and wild type littermates (WT) infected with 1 × 10^5^ PFU of the BeAn strain of Theiler’s murine encephalomyelitis virus. Weight analysis (**A**) and Rotarod performance tests (**B**) showed a continuous increase in body weight and no deterioration in motor coordination in all mice. Semiquantitative scores of perivascular mononuclear cell infiltrates in the brain (**C**) and spinal cord (**D**) at 4, 14 and 98 days post infection (dpi). Cell infiltrates were analyzed in two complete cross sections of the brain at the levels of the hippocampus and cerebellum and complete cross sections of the cervical, thoracic and lumbar spinal cord. Mild to moderate inflammation was found at 4 and 14 dpi but not at 98 dpi in the brain, whereas mild inflammation was present in the spinal cord of at all investigated time points. No significant differences between KO and WT mice in the clinical and histological data were detected using Mann–Whitney tests. n = 7–10. Box plots with all data points. (**E**) Images demonstrate few perivascular mononuclear cells in the hippocampus and spinal cord of all mice at 4 dpi. Hematoxylin and eosin (HE) staining. Bars = 400 µm.

**Figure 2:**
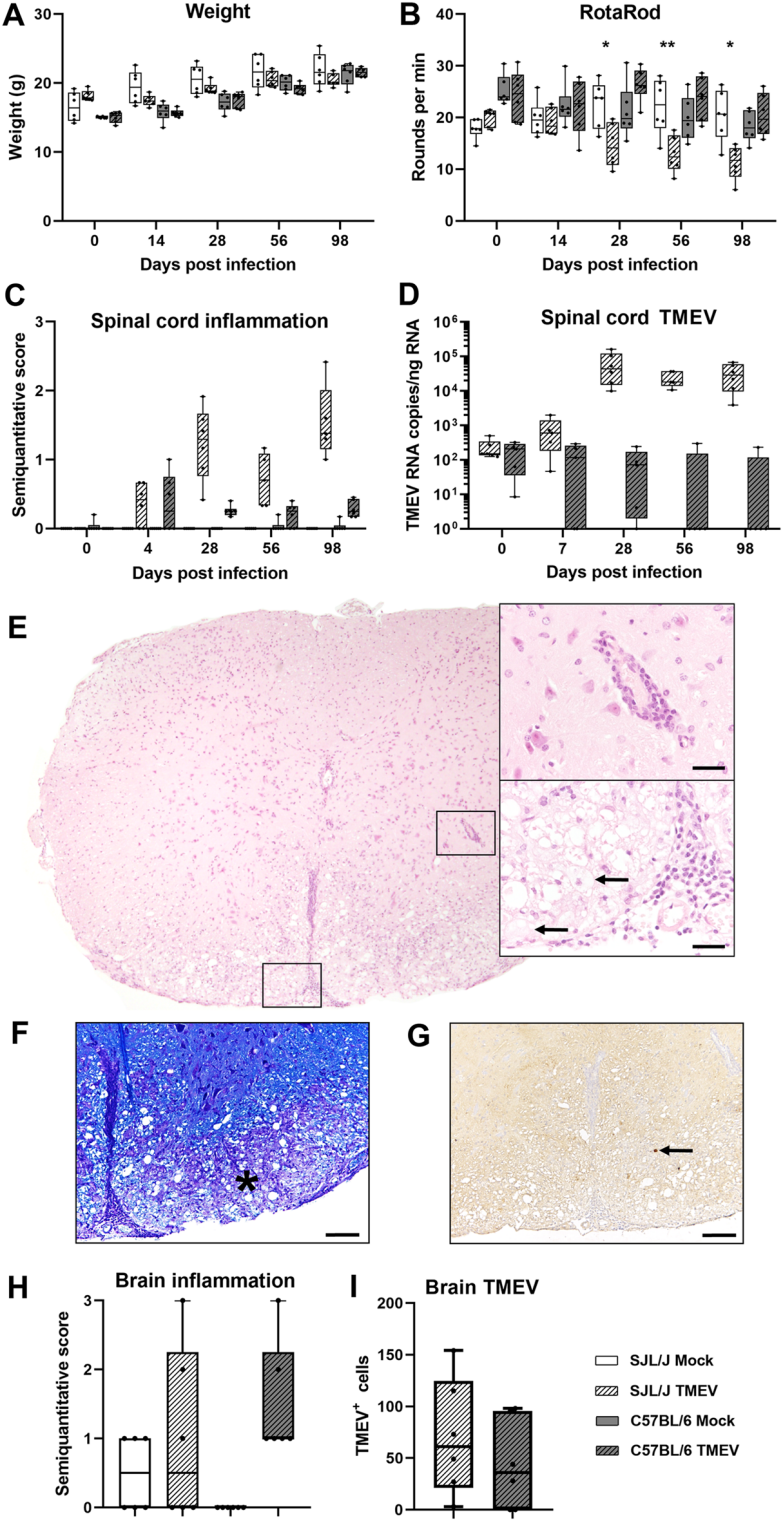
Clinical disease and histological lesions in SJL/J and C57BL/6 mice mock-infected with cell culture medium or infected with 1.63 × 10^6^ PFU of the BeAn strain of Theiler’s murine encephalomyelitis virus (TMEV). Weight analysis (**A**) and Rotarod performance tests (**B**) of TMEV-infected SJL/J showed a continuous increase in body weight but an impaired motor coordination at 28, 56, and 98 days post infection (dpi). No clinical signs were present in C57BL/6 mice. Mann–Whitney tests: *P < 0.05; **P < 0.01. n = 6. (**C**) Semiquantitative scores of perivascular mononuclear cell infiltrates analyzed in complete cross sections of the cervical, thoracic and lumbar spinal cord. Severe inflammation only develops in SJL/J mice. n = 5–6. (**D**) TMEV RNA copy numbers in the spinal cord quantified with RT-qPCR. Note the logarithmic scale and high versus low/no virus replication in SJL/J and C57BL/6 mice at 28, 56 and 98 dpi, respectively. n = 5–6. (**E**) Mononuclear cell infiltrates in the cervical spinal cord of a TMEV-infected SJL/J mouse at 98 dpi. Note perivascular mononuclear cells and gitter cells (arrows) in higher magnifications. Hematoxylin and eosin (HE) staining. Bars = 30 µm. (**F**) Demyelination in the inflamed ventrolateral funiculus of the spinal cord (same SJL/J mouse as in (F)). Note loss of luxol fast blue (LFB) staining due to severe inflammation (asterisk). Bar = 100 µm. (**G**) Immunohistochemistry was used to detect virus antigen in the spinal cord (same mouse as in (E) and (F)). Note TMEV^+^ cell (arrow) in the spinal cord white matter lesion. Avidin–biotin-complex (ABC) method using 3,3'-diaminobenzidine (DAB) as chromogen. Bar = 100 µm. (**H**) Semiquantitative scores of perivascular mononuclear cell infiltrates in the brain analyzed in a complete cross section of the cerebrum at the level of the hippocampus at 4 dpi. Moderate to strong inflammatory lesions were found in TMEV-infected SJL/J and C57BL/6 mice. (**I**) Immunohistochemistry was used to detect virus antigen in a complete cross section of the cerebrum at the level of the hippocampus at 4 dpi. No significant differences in the number of TMEV^+^ cells were found between SJL/J and C57BL/6 mice at 4 dpi using Mann–Whitney tests. n = 6. Box plots with all data points.

Clinical signs were absent in all investigated mice after TMEV infection. Moreover, mice continuously gained weight during the investigation period and their motor coordination did not deteriorate (Fig. 1A,B). *Asc*^−/−^ and *Casp1*^−/−^ mice developed mild to moderate inflammatory brain and spinal cord lesions in the acute phase of the disease (4 and 14 dpi), which did not differ significantly from their wild type littermates (Fig. 1C–E). In the chronic phase of the disease (98 dpi) perivascular mononuclear cell infiltrates were also found in the spinal cord of *Asc*^−/−^ and *Casp1*^−/−^ mice as well as brain of *Asc*^−/−^ mice but not in the brain of *Casp1*^−/−^ mice (Fig. 1C,D). To illustrate a productive virus infection leading to demyelinating lesions in susceptible SJL but not in resistant B6 mice, clinical, histological, and RT-qPCR data of TMEV- and mock-infected SJL and B6 mice are summarized in Fig. 2.

### Immunohistochemistry

**Figure 3:**
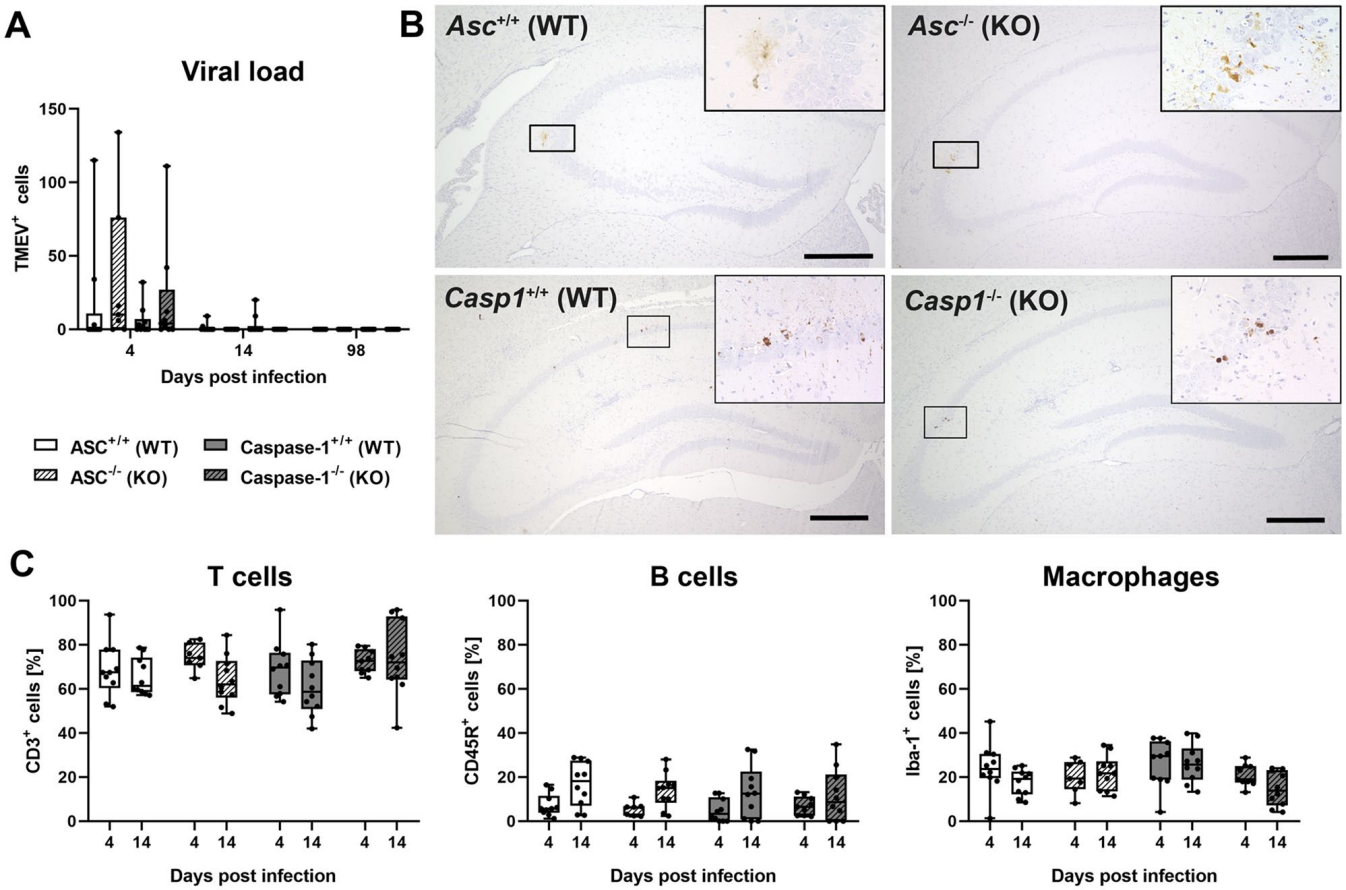
Immunohistochemistry was used to detect virus antigen and determine percentages of perivascular immune cells in the brain of *Asc*^−/−^ and *Casp1*^−/−^ mice (KO) and wild type littermates (WT) infected with 1 × 10^5^ PFU of the BeAn strain of Theiler’s murine encephalomyelitis virus (TMEV). (**A**) All TMEV^+^ cells were counted in a complete cross section of the cerebrum at the level of the hippocampus at 4, 14 and 98 days post infection (dpi). No significant differences in the number of TMEV^+^ cells were found between KO and WT mice using Mann–Whitney tests demonstrating rapid virus elimination. n = 7–10. (**B**) Images show TMEV^+^ cells in the CA1 and CA2 area of the hippocampus of KO and WT mice at 4 dpi. Avidin–biotin-complex (ABC) method using 3,3'-diaminobenzidine (DAB) as chromogen. Bars = 400 µm. (**C**) Percentages of perivascular CD3^+^ T cell, CD45R^+^ B cells and Iba-1^+^ macrophages at 4 and 7 dpi. No significant differences between KO and WT mice were detected using Mann–Whitney tests. n = 10 except *Casp1*^−/−^ mice at 4 dpi (n = 9). Box plots with all data points.

TMEV^+^ cells were mainly detected at 4 dpi in all investigated mouse groups (Fig. 3A,B). Virus antigen was only present in few cells of single animals at 14 dpi and not found at 98 dpi. The perivascular mononuclear cell infiltrates contained approximately 70% CD3^+^ T cells, 10% CD45R^+^ B cells and 20% Iba-1^+^ macrophages (Fig. 3C). No significant differences between *Asc*^−/−^ and *Casp1*^−/−^ mice and their respective wild type littermates were found in the composition of these leukocytes in the perivascular area.

### RT-qPCR

**Figure 4:**
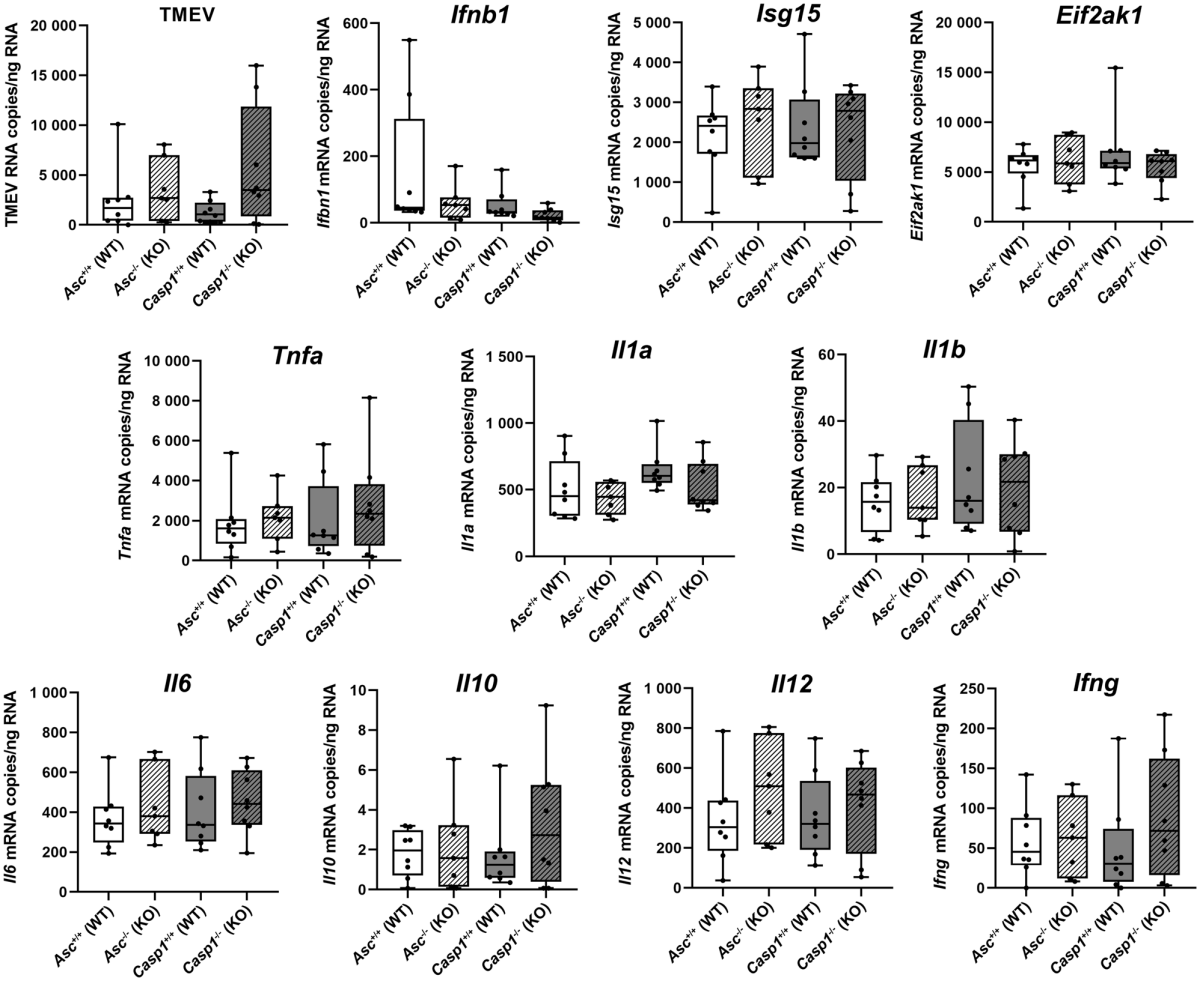
The number of Theiler’s murine encephalomyelitis virus (TMEV) RNA copies/ng RNA as well as *Ifnb1*, *Isg15*, *Eif2ak1* (PKR), *Tnfa*, *Il1a*, *Il1b*, *Il6*, *Il10*, *Il12* (p40) and *Ifng* mRNA copies /ng RNA was quantified in the brain of *Asc*^−/−^ and *Casp1*^−/−^ mice (KO) and wild type littermates (WT) infected with 1 × 10^5^ PFU of the BeAn strain of TMEV at 4 days post infection using RT-qPCR. No significant differences between *Asc*^−/−^ and *Casp1*^−/−^ mice and their respective wild type littermates were found using Mann–Whitney tests. n = 8 (*Asc*^+/+^, *Casp1*^+/+^ and *Casp1*^−/−^ mice), n = 7 (*Asc*^−/−^ mice). Box plots with all data points.

RT-qPCR did not reveal significant differences in the amount of TMEV RNA as well as *Ifnb1*, *Isg15*, *Eif2ak1* (PKR), *Tnfa*, *Il1a*, *Il1b*, *Il6*, *Il10*, *Il12* (p40) and *Ifng* transcript numbers in the brain of *Asc*^−/−^ and *Casp1*^−/−^ mice and their respective wild type littermates at 4 days after TMEV infection (Fig. 4). No *Il17* mRNA was found in the brain of these mice at this time point.

### Western Blot

**Figure 5:**
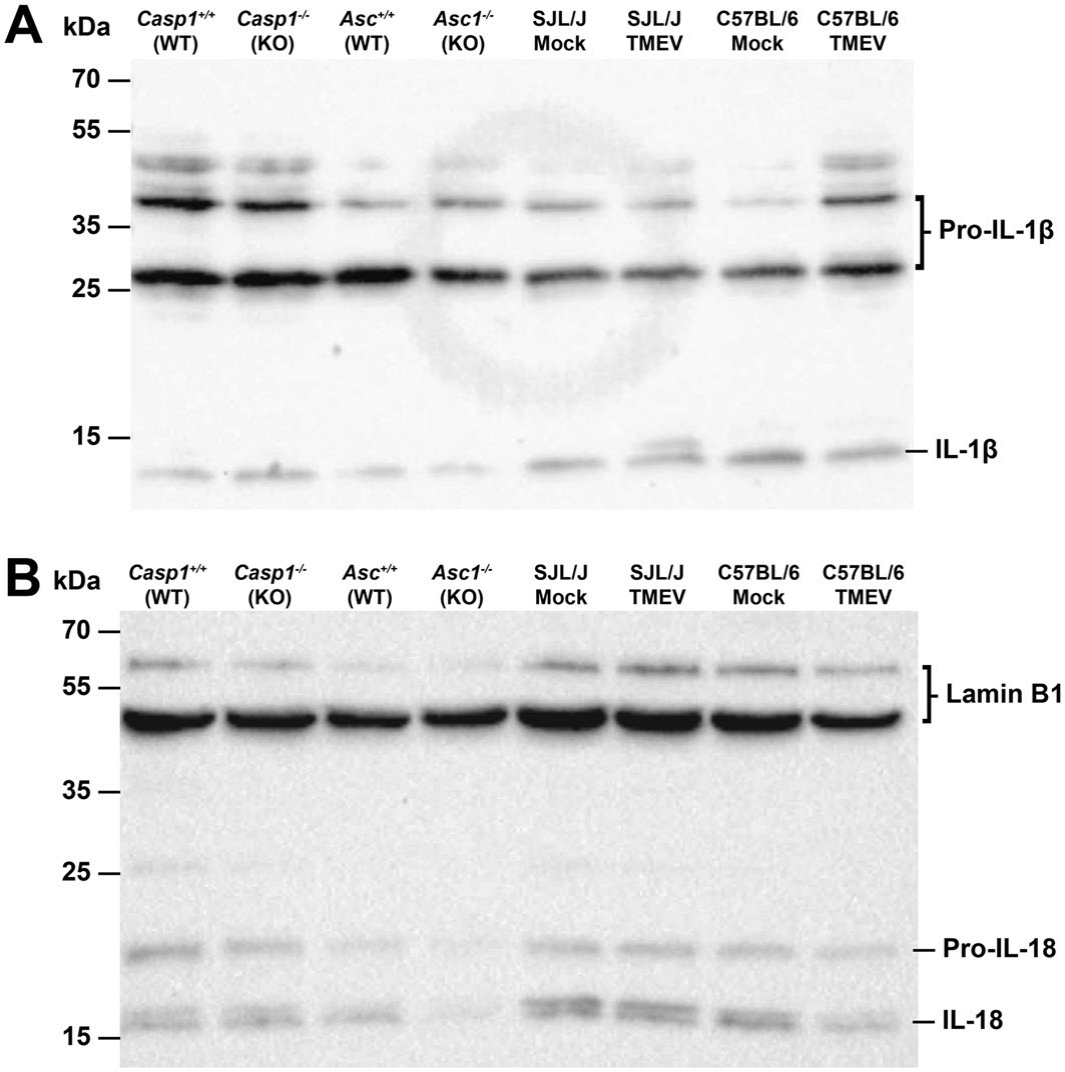
Western Blot was used to detect cleavage of the inactive precursors of the proinflammatory cytokines in the brain of *Asc*^−/−^ and *Casp1*^−/−^ mice (KO) and wild type littermates (WT) infected with 1 × 10^5^ PFU of the BeAn strain of Theiler’s murine encephalomyelitis virus (TMEV). Brain samples of SJL/J and C57BL/6 mice mock-infected with cell culture medium or infected with 1.63 × 10^6^ PFU of the TMEV-BeAn were also included. (**A**) A cleavage of the precursor Pro-IL-1β to the mature IL-1β protein was found in all brain samples. (**B**) Similarly, a cleavage of the precursor Pro-IL-18 to the mature IL-18 protein was demonstrated in all brain samples despite low protein levels in *Asc*^−/−^ mice. Detection of Lamin B1 served as the loading control.

Western blot analysis confirmed the expression of IL-1β and IL-18 proteins in the brain of TMEV-infected mice and wild type littermates at 4 dpi. Moreover, activation/cleavage of the precursor forms of these proinflammatory cytokines (Pro-IL-1β and Pro-IL-18) into the mature proteins was demonstrated in *Casp1*^+/+^, *Casp1*^−/−^, *Asc*^+/+^ and *Asc*^−/−^ mice as well as TMEV- and mock-infected SJL and B6 mice (Fig. 5, Suppl. Figure 1).

## Discussion

ASC and caspase-1 are major components of the inflammasome pathway, which plays a critical role in several neuroinflammatory and neurodegenerative diseases including MS^17,22,31,42^. Inflammasome activation induces the maturation of the proinflammatory cytokines IL-1β, IL-18, and IL-33 by proteolytic cleavage of their precursor forms. IL-1β is involved in the acute phase response and stimulates the expression of adhesion molecules on endothelial cells and chemokines necessary for leukocyte recruitment. IL-1β also orchestrates the differentiation and function of innate and adaptive lymphoid cells^43^. For instance, the induction of eicosanoids such as PGE2 by IL-1β limits immunosuppressive effects of IFN-I on T cell functions^26,44^. IL-18 has been implicated in several autoimmune diseases including MS, because it stimulates γδ T cells to express IL-17 and promotes the production of the Th1 cytokine IFNγ from T cells and NK cells^45^. IL-33 is a potent inducer for a Th2 immune response, but this IL-1-like cytokine is inactivated after maturation by caspase-1^46,47^.

However, the present study revealed no consequences of ASC or caspase-1 deficiency on virus elimination, CNS inflammation, cytokine and IFN-I expression and clinical outcome in TMEV-infected B6 mice. In contrast, the infection of immunodeficient IFN-β knockout mice (also B6 background) with the same virus strain and dosage resulted in virus persistence, increased cytokine expression and mild inflammatory demyelinating spinal cord lesions^11^. Lack of inflammasome-dependent IL-1β maturation might have been compensated by increased expression of IL-1α, which can be upregulated in TMEV-infected astrocytes and bind to the same receptor^48–50^. Nonetheless, no significant differences in *Il1a* mRNA levels were found between ASC- and caspase-1-deficient mice and their wild type littermates. Moreover, *Tnfa*, *Il6*, *Il10*, *Il12*, and *Ifng* mRNA levels were similar in all investigated mice at 4 dpi indicating that ASC- and caspase-1-deficiency does not influence cytokine expression in the brain of TMEV-infected B6 mice significantly.

Interestingly, Western blot analysis demonstrated the activation/cleavage of IL-1β and IL-18 proteins in both knockout mouse strains, which can be explained by alternative inflammasome-independent pathways of IL-1β and IL-18 maturation. In addition to microorganisms such as *Candida albicans*, *Staphylococcus aureus* and *Streptococcus pyogenes*, monocyte/macrophages, neutrophils, NK cells, mast cells and epithelial cells can produce proteases able to cleave pro-IL-1β including the serine proteases proteinase 3, cathepsin G, elastase, granzyme A, chymase, chymotrypsin as well as the metalloproteinases Meprin α and Meprin β^16,51^. Correspondingly, an increased expression of cathepsins and granzymes, which are released by monocytes/macrophages and NK cells respectively, has been described in the brain and spinal cord of TMEV-infected mice^6,52^. Consequently, the production of different serine proteases by NK cells and microglia/macrophages likely compensates lack of inflammasome-dependent regulation of IL-1-family cytokines during TMEV infection.

In conclusion, ASC or caspase-1 deficiency had no effect on virus elimination, CNS inflammation, IFN-I and cytokine gene expression and clinical outcome in TMEV-infected B6 mice. The present results demonstrate that inflammasome-dependent pathways do not play a major role in the resistance of B6 mice to TMEV-IDD due to the activity of other proteases able to cleave IL-1β, IL-18, and IL-33. Nevertheless, future studies are needed to unravel the complex interactions of the inflammasome and other proinflammatory pathways in neuroinflammatory diseases.

## Materials and methods

### Animal experiment

*Asc*^−/−^ (B6.129S5-Pycard^tm1Flv^) mice (http://www.informatics.jax.org/allele/MGI:3686870) and *Casp1*^−/−^ (Casp1^tm2.1Flv^) were generated as described^53–56^. Briefly, *Asc* and *Casp1* targeting vectors were electroporated into 129SvEvBrd Lex-1 and JM8 C57BL/6 embryonic stem cells, respectively. Offspring was backcrossed to B6 mice. Heterozygous knockout mice were used to breed 27 *Asc*^−/−^ and 28 *Casp1*^−/−^ mice and 60 wild type littermates. SJL/J and B6 mice were obtained from Harlan Winkelmann (now Envigo RMS GmbH, Düsseldorf, Germany). Mice were kept under controlled environmental conditions (22–24 °C; 50–60% humidity; 12/12 h light/dark cycle) with free access to standard rodent diet (R/M-H; sniff Spezialdiäten GmbH, Soest, Germany) and tap water. At the age of five to six weeks, groups of seven to ten knockout mice and wild type littermates were inoculated into the right cerebral hemisphere with 1 × 10^5^ plaque-forming units (PFU) per mouse of the BeAn strain of TMEV in 20 µl Dulbecco’s modified Eagle medium (DMEM; PAA Laboratories, Cölbe, Germany) with 2% fetal calf serum and 50 μg/kg gentamicin. This virus stock and dose has been used in several publications and is known to induce TMEV-IDD in SJL mice and in B6 mice^11,39,57–60^. To illustrate a productive viral infection and disease course, data of additional experiments using SJL and B6 mice infected with TMEV-BeAn (1.63 × 10^6^ PFU) or mock-infected with DMEM were included in the study. Inoculation was performed under general anesthesia using an intraperitoneal injection of medetomidine (0.5 mg/kg, Domitor; 50 µl for a mouse of 20 g; Pfizer, Karlsruhe, Germany) and ketamine (100 mg/kg, Ketamin 10%; 100 µl for a mouse of 20 g; WDT eG, Garbsen, Germany). General appearance, activity, and gait was evaluated weekly as previously described^61^. Moreover, a rotarod assay (RotaRod Treadmill, TSE Technical & Scientific Equipment, Bad Homburg, Germany) was performed every week to test motor strength and control of TMEV-infected mice^62^. Animals were sacrificed at 4, 14 and 98 dpi using an intraperitoneal injection of an overdose of medetomidine (1 mg/kg, Domitor; 20 µl for a mouse of 20 g; Pfizer) and ketamine (200 mg/kg, Ketamin 10%; 40 µl for a mouse of 20 g; WDT eG). Segments of the brain and spinal cord of mice were removed immediately after death and either formalin-fixed and paraffin-embedded or snap-frozen in OCT embedding compound (Sakura Finetek Europe, Zoeterwoude, Netherlands) using liquid nitrogen^63^. Animal experiments were conducted in accordance with the German Animal Welfare Law and the ARRIVE guidelines^64^ and were authorized by the local government (Niedersächsisches Landesamt für Verbraucherschutz und Lebensmittelsicherheit, Oldenburg, Germany, permission numbers: 509c-42502-02/589, 509.6-42502-04/860, 33.12-42502-04-14/1656).

### Histological examination

Two micrometers paraffin sections of brain (cerebrum and cerebellum) and spinal cord (cervical, thoracic and lumbar) were routinely stained with hematoxylin and eosin (HE). The degree of inflammation was evaluated in two complete cross sections of the brain at the levels of the hippocampus and cerebellum and complete cross sections of the cervical, thoracic and lumbar spinal cord using a semiquantitative scoring system of perivascular mononuclear cell infiltrates (0 = normal, 1 = single inflammatory cells, 2 = 2–3 layers of infiltrates, 3 = more than 3 layers of infiltrates surrounding meningeal and parenchymal vessels) after blinding^6,10,61,65^.

### Immunohistochemistry

Immunohistochemistry was performed as previously described^6,10,66–68^. Briefly, paraffin sections were blocked with 20% goat serum and stained with rabbit polyclonal antibodies directed against TMEV antigen (VP1; 1:2000), CD3 (T lymphocytes; Agilent Technologies Deutschland GmbH, Waldbronn, Germany; A045201; 1:500) and Iba-1 (microglia/macrophages; Wako Chemicals GmbH, Neuss, Germany; 019-19741; 1:1000) or rat monoclonal antibody directed against CD45R (B lymphocytes; BD Biosciences, Heidelberg, Germany; clone RA3-6B2; 553085; 1:1000) overnight at 4 °C. Primary antibodies were replaced by rabbit serum (R4505, Merck KGaA, Darmstadt, Germany) or rat serum (R9759; Merck KGaA) as negative control. Biotinylated goat-anti-rabbit IgG (BA-1000, Vector Laboratories, Burlingame, CA, USA; 1:200) and rabbit-anti rat IgG (BA-4001; Vector Laboratories; 1:200) were used as secondary antibodies. Immunolabeling was visualized by the avidin–biotin-peroxidase complex (ABC) method (PK-6100, Vector Laboratories) with 3,3-diaminobenzidine (DAB, Merck KGaA) as substrate and slight counterstaining was performed using Mayer’s hematoxylin. Percentages of perivascular CD3^+^ T cells, CD45R^+^ B cells and Iba-1^+^ macrophages were determined by randomly counting 100 cells in the Virchow-Robin space of vessels present in one cross section of the cerebrum at the level of the hippocampus. Moreover, all TMEV^+^ cells were counted in a serial section at this level.

### Polymerase chain reaction

RNA has been isolated from a complete cross section of the OCT-embedded cerebrum at the level of the hippocampus. Real-time quantitative polymerase chain reaction (RT-qPCR) was performed for TMEV, *Ifnb1*, *Isg15*, *Eif2ak1*, *Tnfa*, *Il1a*, *Il1b*, *Il6*, *Il10*, *Il12* (p40), *Il17*, *Ifng* and three housekeeping genes (*Actb*, *Gadph*, *Hprt1*) using standard protocols, the AriaMx Real-Time PCR system (Agilent Technologies Deutschland GmbH), and Brilliant III Ultra-Fast SYBR® QPCR Master Mixes as described^49,66,68,69^. Tenfold serial dilution standards were used to quantify the results. Experimental data were normalized using a normalization factor calculated from the three housekeeping genes^70^. Specificity of each reaction was controlled by melting curve analysis.

### Western blot

Proteins have been isolated from a complete cross section of the OCT-embedded cerebrum at the level of the hippocampus. Cells were lysed in radioimmunoprecipitation assay (RIPA) buffer (1 mM phenylmethylsulfonyl fluoride, 1% sodium deoxycholate, 50 mM Tris–HCl [pH 7.4], 1% Triton X-100, 0.1% SDS, 150 mM NaCl) with added protease inhibitor cocktail (cOmplete, EDTA free; Roche Diagnostics GmbH, Mannheim, Germany). The total protein concentration of each sample was determined using the Pierce™ BCA™ Protein-Assay (Thermo Fisher, Rockford, IL, USA) according to the manufacture’s instructions. 20 µg of total protein were separated by SDS–polyacrylamide gel electrophoresis followed by transfer to polyvinylidene difluoride (PVDF) membranes (Novex™). Rabbit polyclonal antibodies were used to detect IL-1β (P420B; Thermo Fisher; 1:1000), IL-18 (PA5-79481; Thermo Fisher; 1:1000), and Laminin (PA5-19468; Thermo Fisher; 1:1000). Then, blots were incubated with a peroxidase-coupled goat anti-rabbit secondary antiserum (32460; Thermo Fisher; 1:500) and the binding was visualized using SuperSignal™ West Femto Maximum Sensitivity Substrate (34095; Thermo Fisher) and a ChemiDoc™ MP Imaging System (BioRad). For band size determination PageRuler™ Plus Prestained Protein Ladder (10 to 250 kDa; 26620; Thermo Fisher) was used.

### Statistical analysis

Statistical analysis was performed using Prism 6 (GraphPad Software, La Jolla, CA, USA). Mann–Whitney tests were used to compare clinical, histological, immunohistochemical and PCR data (knockout vs. wild type mice). *P* < 0.05 was considered as statistical significant.

## Supplementary Information

Supplementary Figure 1.

## Data Availability

The datasets generated and analyzed during the current study can be obtained from the corresponding author on reasonable request.
