# Supplementary material for: ASC- and caspase-1-deficient C57BL/6 mice do not develop demyelinating disease after infection with Theiler’s murine encephalomyelitis virus
Source: Sci Rep. 2023 Jul 6;13:10960. doi: 10.1038/s41598-023-38152-3 (PMC10326010; doi:10.1038/s41598-023-38152-3)
Supplement: Supplementary file 1 — Supplementary Figure 1. [file 41598_2023_38152_MOESM1_ESM.docx]

**Supplementary Information**

**ASC- and caspase-1-deficient C57BL/6 mice do not develop demyelinating disease after infection with Theiler’s murine encephalomyelitis virus**

D. Li, M. Bühler, S. Runft, G. Gerold, K. Marek, W. Baumgärtner, T. Strowig, I. Gerhauser

**Suppl. Figure 1:** Western Blot was used to detect cleavage of the inactive precursors of the proinflammatory cytokines in the brain of *Asc*^-/-^ and *Casp1*^-/-^ mice (KO) and wild type littermates (WT) infected with 1×10^5^ PFU of the BeAn strain of Theiler’s murine encephalomyelitis virus (TMEV). Brain samples of SJL/J and C57BL/6 mice mock-infected with cell culture medium or infected with 1.63×10^6^ PFU of the TMEV-BeAn were also included. (**A**) A cleavage of the precursor Pro-IL-1β to the mature IL-1β protein was found in all brain samples. (**B**) Similarly, a cleavage of the precursor Pro-IL-18 to the mature IL-18 protein was demonstrated in all brain samples despite low protein levels in *Asc*^-/-^ mice. Detection of Lamin B1 served as the loading control.
